# Conditioned Medium from Human Amnion-Derived Mesenchymal Stem Cells Regulates Activation of Primary Hepatic Stellate Cells

**DOI:** 10.1155/2018/4898152

**Published:** 2018-10-08

**Authors:** Qingjie Fu, Shunsuke Ohnishi, Naoya Sakamoto

**Affiliations:** Department of Gastroenterology and Hepatology, Hokkaido University Graduate School of Medicine, Sapporo, Japan

## Abstract

Mesenchymal stem cells (MSCs), or multipotent mesenchymal stromal cells, are present in almost all organs and tissues, including the amnion. Human amnion-derived mesenchymal stem cell (hAMSC) transplantation has been reported to ameliorate liver fibrosis in animal models. However, the mechanism for the prevention of liver fibrosis is poorly understood. In this study, we investigated the effects, and underlying mechanisms, of a conditioned medium obtained from hAMSC cultures (hAMSC-CM) on a primary culture of rat hepatic stellate cells (HSCs). We observed that in routine culture, hAMSC-CM in HSCs significantly inhibited the expression of alpha-smooth muscle actin (α-SMA), an activation marker of HSCs, and the production of collagen type 1 (COL1), a dominant component of the extracellular matrix (ECM) in the culture medium. In addition, hAMSC-CM upregulated the expression of ECM degradation-related genes, such as metalloproteinase- (*Mmp*-) 2, *Mmp*-9, *Mmp*-13, and tissue inhibitor of metalloproteinase- (*Timp*-) 1; however, it did not affect the expression of collagen type 1α1 (*Col1a1*). These regulatory effects on HSCs were concentration-dependent. A cell proliferation assay indicated that hAMSC-CM significantly suppressed HSC proliferation and downregulated the expression of cyclin B (*Ccnb*), a proliferation-related gene. Transforming growth factor-beta (TGF-β) treatment further activated HSCs and hAMSC-CM significantly inhibited the upregulation of *α-Sma* and *Col1a1* induced by TGF-β. These findings demonstrated that hAMSC-CM can modulate HSC function via secretory factors and provide a plausible explanation for the protective role of hAMSCs in liver fibrosis.

## 1. Introduction

Liver fibrosis, the precursor to cirrhosis, is a complex inflammatory and fibrogenic condition caused by chronic liver injury and an imbalance in extracellular matrix (ECM) synthesis and degradation mediated primarily by activated hepatic stellate cells (HSCs) [1]. Following liver injury, HSCs undergo an activation process and transform from quiescent, vitamin A-storing cells into highly proliferative, myofibroblast-like cells, upregulating collagen synthesis, especially collagen type 1*α*1 (COL1A1), and expressing alpha-smooth muscle actin (α-SMA), an activation marker of HSCs [2–4]. In addition, activated HSCs regulate ECM components by producing matrix metalloproteinases (MMPs), such as MMP-2, MMP-9, and MMP-13, and tissue inhibitors of metalloproteinases (TIMPs), such as TIMP-1 and TIMP-2 [1].

Mesenchymal stem cells (MSCs) are stromal cells that exhibit multilineage differentiation and self-renewal ability [5]. In addition, a variety of animal models as well as clinical trials have demonstrated the anti-inflammatory, antifibrotic, and antiapoptotic effects of MSCs in either MSC transplantation or MSC conditioned medium (CM) administration [6]. Although MSCs were first reported to be derived from bone marrow, they have been isolated from almost all tissues, including adipose tissue, the umbilical cord, dental pulp, and the amnion [7]. The advantages of human amnion-derived mesenchymal stem cells (hAMSCs) are that (i) they can be obtained in large numbers without invasive procedures and (ii) they have enormous proliferative capacity [8]. Therefore, hAMSCs have attracted much attention in the cell therapy and regenerative medicine fields [9]. In a previous study, we found that hAMSC transplantation ameliorated liver fibrosis in rats, possibly through secretory factors from hAMSCs [10]. Thus, in this study, we investigated the effect of a conditioned medium obtained from hAMSC cultures (hAMSC-CM) on primary HSCs and also the underlying mechanisms of its antifibrotic effect *in vitro*.

## 2. Materials and Methods

### 2.1. Animals

The Animal Care and Use Committees of Hokkaido University approved the experimental protocol and animal care. Male Sprague–Dawley (SD) rats (400–450 g in weight) were procured from Japan SLC (Hamamatsu, Japan). They were housed in a temperature-controlled room (24°C) on a 12-hourly light–dark cycle and were provided with standard chow and water *ad libitum* until the time of the study.

### 2.2. Isolation and Expansion of hAMSCs

The Medical Ethical Committee of Hokkaido University, Graduate School of Medicine, Sapporo, Japan, approved the study. A pregnant woman provided written informed consent for use of her fetal membrane, which was obtained during her cesarean delivery. Isolation and expansion of hAMSCs were performed as described in a previous study [11]. The expanded hAMSCs were stored in liquid nitrogen until use.

### 2.3. Preparation of hAMSC-CM

We recovered cryopreserved hAMSCs and cultured them until the cells reached a subconfluent state (passage 6). After washing them with Hank's balanced salt solution without calcium, magnesium, or phenol red (HBSS (−); Nacalai Tesque, Kyoto, Japan), we further cultured the cells with serum-free minimum essential medium alpha (MEM*α*; Nacalai Tesque) for 48 h. Next, we collected hAMSC-CM and removed the debris by centrifugation at 1120 × *g* for 5 min. Serum-free MEM*α* incubated in a cell-free dish for 48 h was used as a standard medium (SM), and both SM and hAMSC-CM were stored at −80°C until use.

### 2.4. Isolation and Purification of HSCs

Previous studies have shown various methods of isolating HSCs [12–15]; we performed isolation after modifying several steps. The SD rats were anesthetized by intraperitoneal injection of 6.48 mg/100 g body weight of pentobarbital sodium (Kyoritsu Seiyaku, Tokyo, Japan). Each rat's liver was perfused via the portal vein using an 18 G needle (Terumo, Tokyo, Japan) that was fixed by sutures. Buffers were preheated to 42°C and pumped into the liver using a peristaltic pump.

#### 2.4.1. Liver Perfusion and Enzymatic Digestion

Initially, the liver was perfused with 60 mL of HBSS (−) containing 1 mM ethylenediaminetetraacetic acid (EDTA; Thermo Fisher Scientific, Waltham, MA, USA) at 18 mL/min, and when the liver became distended, the inferior vena cava (IVC) was cut. We incised the diaphragm and clipped the intrathoracic IVC with a vascular clamp to ensure the buffers were drained completely via the abdominal IVC incision. Next, the liver was infused with 200 mL of HBSS (−) supplemented with 100 U/mL of collagenase II (Worthington Biochemical, Lakewood, NJ, USA) at 7.5 mL/min. The perfused liver was removed, minced using two tweezers in a sterile dish containing HBSS (−), and further digested in a flask containing 65 U/mL of collagenase II and 1% (*v*/*v*) deoxyribonuclease I (DNase I; Worthington Biochemical) with an initial concentration of 40 kU/mL. The flask was placed on a stir plate and shaken at 70 rpm/min for 20 min in an incubator at 37°C. The resulting cell suspension was filtered using first a 100 μm and then a 70 μm cell strainer and was centrifuged for 10 min at 600 × *g* and 4°C. The pellet was washed and resuspended using HBSS (−) containing 120 μL of DNase I and then centrifuged for 3 min at 50 × *g* and 4°C. Then, the supernatant was collected and centrifuged for 10 min at 400 × *g* and 4°C.

#### 2.4.2. Density Gradient Centrifugation

The pellet was resuspended in HBSS (−) containing 120 μL of DNase I and, then, was mixed with Percoll (GE Healthcare Bio-Sciences, Uppsala, Sweden) to a final concentration of 30% (*v*/*v*) at 20°C. Next, 10 mL of a thoroughly mixed cell–Percoll suspension was pipetted into a 15 mL centrifugation tube, and 2 mL of HBSS (−) was gently overlaid on the suspension. Centrifugation was performed at 1470 × *g* and 20°C for 25 min with slow acceleration and deceleration. The interphase containing enriched HSCs between HBSS (−) and the 30% Percoll layer was harvested and washed using HBSS (−) for 8 min at 400 × *g* and 4°C.

#### 2.4.3. Fluorescence-Activated Cell Sorting (FACS) for HSCs

The HSC pellet was resuspended in phenol red-free MEM*α* supplemented with 1.5% fetal bovine serum (FBS; Thermo Fisher Scientific, Carlsbad, LA, USA), and the suspension was filtered using a 40 μm cell strainer and adjusted to 6–8 × 10^6^ cells/mL. A BD FACS Aria III Cell Sorter (BD Biosciences, San Jose, CA, USA) was used to perform HSC sorting. We used endogenous retinoid fluorescence of HSCs as a selection marker, performed excitation via a 375 nm laser, and measured the emission using a 450/20 nm band-pass filter at a Hoechst-blue channel. We used a 100 μm nozzle and a 2.0 neutral density filter, and the sample loading port was set to 4°C, 300 rpm. The sorting mode was set up in purity mode, and 2 μL/10^6^ cells of 7-aminoactinomycin D solution (7-AAD; BD Biosciences) was added to the suspension immediately before sorting. The 15 mL collection tube was made of polypropylene and was coated with FBS overnight at 4°C. It contained 10 mL of Dulbecco minimal essential medium (DMEM; Nacalai Tesque) supplemented with 17% FBS. After sorting, the cells were centrifuged for 5 min at 400 × *g* and 4°C and cultured. Their purity was determined by flow cytometry with autofluorescence.

### 2.5. Culture Models

All the cells were cultured in a humidified atmosphere of 95% air and 5% CO_2_ at 37°C. All the culture media were supplemented with 100 U/mL of penicillin and 100 μg/mL of streptomycin. A Luna automated cell counter (Logos Biosystems, Anyang, South Korea) was used to take a cell count.

#### 2.5.1. Routine Culture of HSCs

Approximately 5 × 10^4^ HSCs were seeded on 12-well plastic plates, on which the cells were automatically activated and proliferated [16]. They were cultured in 2 mL of stellate cell medium (SteCM; ScienCell, Carlsbad, CA, USA) supplemented with 2% FBS (ScienCell) and stellate cell growth supplement (SteCGS; ScienCell) for 48 h. Then, the HSCs were washed thrice using HBSS (−) and were cultured for 48 h with SM or hAMSC-CM. In addition, hAMSC-CM was mixed separately with the SM in two concentrations: 50% and 25% (*v*/*v*) of hAMSC-CM. These different concentrations of hAMSC-CM were also used for culturing washed HSCs for 48 h as described above.

#### 2.5.2. Transforming Growth Factor-Beta (TGF-β) Treatment

Approximately 5 × 10^4^ HSCs were cultured in 12-well plates in SteCM containing 2% FBS and SteCGS for 48 h and washed thrice using HBSS (−). Subsequently, the HSCs were treated with SM or hAMSC-CM supplemented with 5 ng/mL of transforming growth factor-beta 1 (TGF-β1; R&D Systems, Minneapolis, MN, USA) for 48 h. HSCs cultured in SM or hAMSC-CM served as negative controls.

### 2.6. Flow Cytometry Analysis of hAMSCs

Following the manufacturer' instructions, we harvested cultured hAMSCs with 0.5% trypsin/EDTA and stained them using a Human MSC Analysis Kit (BD Biosciences) containing phycoerythrin- (PE-) conjugated anticluster of differentiation 44 (anti-CD44), allophycocyanin- (APC-) conjugated anti-CD73, fluorescein-isothiocyanate- (FITC-) conjugated anti-CD90, and PerCP-Cy5.5-conjugated anti-CD105 antibodies, as well as a negative mixture comprising PE-conjugated anti-CD11b, anti-CD19, anti-CD34, anti-CD45, and anti-human leukocyte antigen–antigen D-related (HLA-DR) antibodies. All the cells were resuspended in HBSS (−), filtered using a 40 μm strainer, and then analyzed using a BD FACSCanto II Flow Cytometer (BD Biosciences).

### 2.7. Immunofluorescent Staining

HSCs cultured in SM and hAMSC-CM were washed thrice using HBSS (−), fixed in methanol for 10 min at 4°C, and then incubated in anti-rat α-SMA (1:500; Abcam, Cambridge, UK) in HBSS (−) containing 2% FBS for 1 h at 4°C. After washing, we incubated the cells in Alexa Flour 488-conjugated secondary antibody (1:1000; Cell Signaling Technology, Danvers, MA, USA) for 30 min at 4°C in the dark. Subsequently, we stained the nucleus with Hoechst 33342 (1:1000; Thermo Fisher Scientific) for 2 min at room temperature. Then, the cells were washed twice and analyzed using a FluoView FV10i confocal laser scanning microscope (Olympus, Tokyo, Japan); all the micrographs were taken under the same exposure time and laser intensity. ImageJ software (http://imagej.nih.gov/ij/) was used to measure fluorescence intensity.

### 2.8. Ribonucleic Acid (RNA) Isolation and Quantitative Reverse-Transcription Polymerase Chain Reaction (qRT-PCR)

RNA of the cultured HSCs was extracted using a RNeasy Mini Kit (Qiagen, Hilden, Germany), and 20 ng of total RNA was reverse-transcribed into complementary deoxyribonucleic acid (cDNA) using a PrimeScript RT Reagent Kit with a genomic deoxyribonucleic acid (gDNA) Eraser (Takara Bio, Kusatsu, Japan) in a Veriti 96-well Thermal Cycler (Applied Biosystems, Waltham, MA, USA), incubated at 37°C for 15 min and 85°C for 15 s. Polymerase chain reaction (PCR) was carried out in a total reaction volume of 25 μL containing 5 μL of template cDNA, 12.5 μL of a Platinum SYBR Green PCR Mix (Invitrogen, Carlsbad, CA, USA), and 2 μL of a 10 μM corresponding primer mixture. PCR conditions, running on a StepOnePlus Real Time PCR System (Applied Biosystems), included predenaturation at 95°C for 20 s followed by 40 cycles at 95°C for 3 s and 60°C for 7 s. A melting curve was created to validate the specificity of the amplification products. A relative expression was determined using the standard curve method with platelet-derived growth factor receptor beta (*Pdgfrb*) used as an endogenous control [14].  Table 1 shows the primer sequences.

### 2.9. Proliferation Assay

Approximately 2 × 10^4^ HSCs were cultured in a 96-well plate with SteCM containing 2% FBS and SteCGS for 48 h. Then, we changed the medium to SM or hAMSC-CM and cultured the cells further for 48 h. HSC proliferation was examined using a Cell Counting Kit-8 (CCK-8; Dojindo Laboratories, Kumamoto, Japan) at 0, 24, and 48 h after changing the medium; the medium without cells was used as a blank control. A GloMax-Multi+ Detection System (Promega, Fitchburg, WI, USA) was used to measure absorbance.

### 2.10. Collagen Type 1 (COL1) Assay

We evaluated the COL1 concentration in SM and hAMSC-CM using a rat COL1 enzyme-linked immunosorbent assay (ELISA) kit (MyBioSource, San Diego, CA, USA) according to the manufacturer's instructions. Cell-free SM and hAMSC-CM were incubated at the same time and taken as blank controls to determine the baseline.

### 2.11. Statistical Analysis

GraphPad Prism 7.0 (GraphPad Software, La Jolla, CA, USA) was used to perform the statistical analysis, and the data were expressed as mean ± standard deviation (SD). Intergroup differences were identified using one-way analysis of variance (ANOVA), followed by the Tukey test. Unpaired *t*-tests or Welch's test was used to identify pairwise differences. The differences were considered statistically significant at *P* < 0.05.

## 3. Results

### 3.1. Characterization of hAMSCs

**Figure 1:**
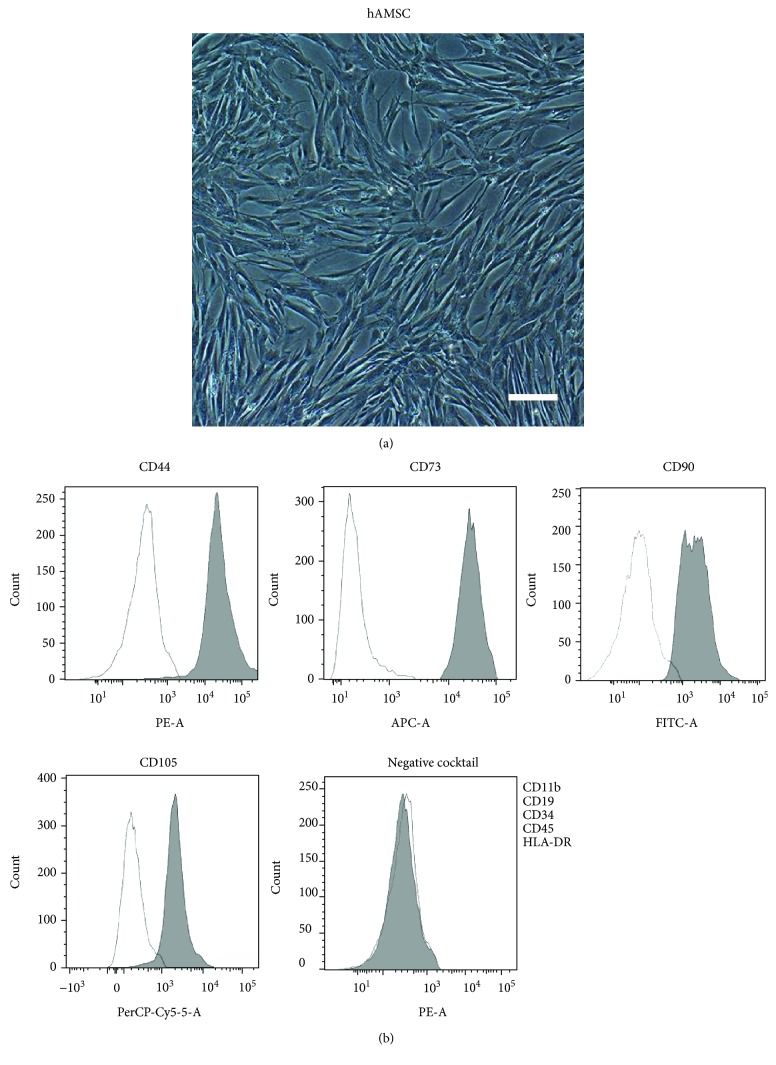
Characterization of cultured hAMSCs. (a) Morphology of cultured hAMSCs. Scale bar = 200 μm. (b) Flow cytometry analysis of cultured hAMSCs.

We observed that cultured hAMSCs have a typical morphology of fibroblast-like cells (Figure 1(a)). Flow cytometry showed that hAMSCs exhibit high expression of CD44, CD73, CD90, and CD105 but no expression of CD11b, CD19, CD34, CD45, or HLA-DR (Figure 1(b)), which is consistent with a characteristic of MSCs [17, 18]. The high expression of MSC-specific marker *ITGA11* and low expression of fibroblast-specific marker *CD26* [19] in cultured hAMSCs indicated that fibroblast contamination is rarely observed in these hAMSCs (Supplementary Figure 1).

### 3.2. Isolation and Characterization of HSCs

**Figure 2:**
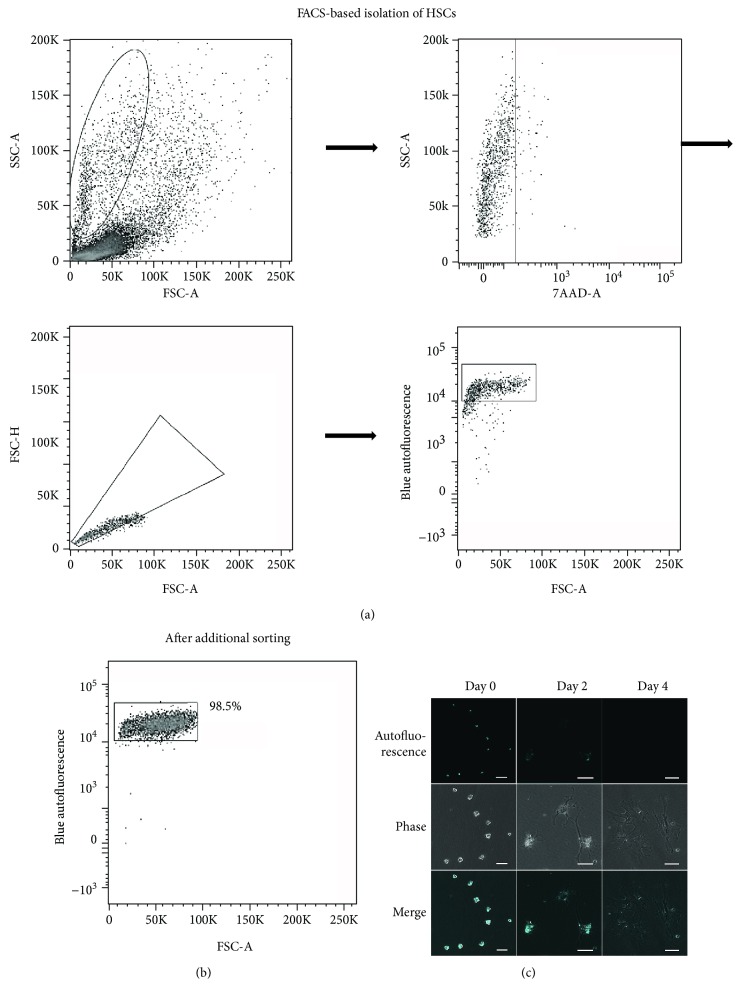
Gating strategy for HSC purification using FACS and HSC characterization. (a) Cells were gated on the basis of their FSC and SSC, viable cells were selected using 7-AAD, and doublets were excluded by FSC-A/FSC-H. Finally, HSCs were selected on the basis of the cyan light emission from retinol. (b) Purity of HSCs after FACS. (c) Primary HSCs are irregularly round-shaped, and retinol-rich lipid droplets emit cyan autofluorescence by a 352 nm laser. The HSCs cultured for 2 days became extended and presented an asteroid phenotype, whereas large numbers of lipid droplets were observed still. No autofluorescence could be detected after culturing HSCs for 4 days, and the cells extended further. FSC: forward scatter; SSC: sideward scatter; 7-AAD: 7-aminoactinomycin D; NUV: near ultraviolet. Scale bar = 50 μm.

First, we gated cells with high sideward scatter (SSC) and low forward scatter (FSC) [20], and 7-AAD was used to select living cells (Figure 2(a)). We used the forward scatter area/forward scatter height (FSC-A/FSC-H) to exclude doublets, and a high autofluorescence area was gated as HSCs (Figure 2(a)). FACS of HSCs resulted in a final purity of >98%, as defined by retinol-based autofluorescence (Figure 2(b)). The freshly isolated HSCs were irregularly round-shaped, and their cytoplasm was rich in lipid droplets. When excited at 352 nm, the vitamin A-rich lipid droplets emitted cyan intrinsic autofluorescence (Figure 2(c)). Postculturing for 2 days, HSCs became extended and presented an asteroid phenotype, accompanied by a reduction of lipid droplets (Figure 2(c)). HSCs were further activated by routine culture, and it was difficult to observe autofluorescence postculturing for 4 days (Figure 2(c)), suggesting that quiescent HSCs were activated by routine culture. Isolated HSCs proliferated well after seeding (Supplementary Figure 2A), and long-term culture showed that HSCs proliferated rapidly with good viability (Supplementary Figure 2B).

### 3.3. Effects of hAMSC-CM on Routinely Cultured HSCs

**Figure 3:**
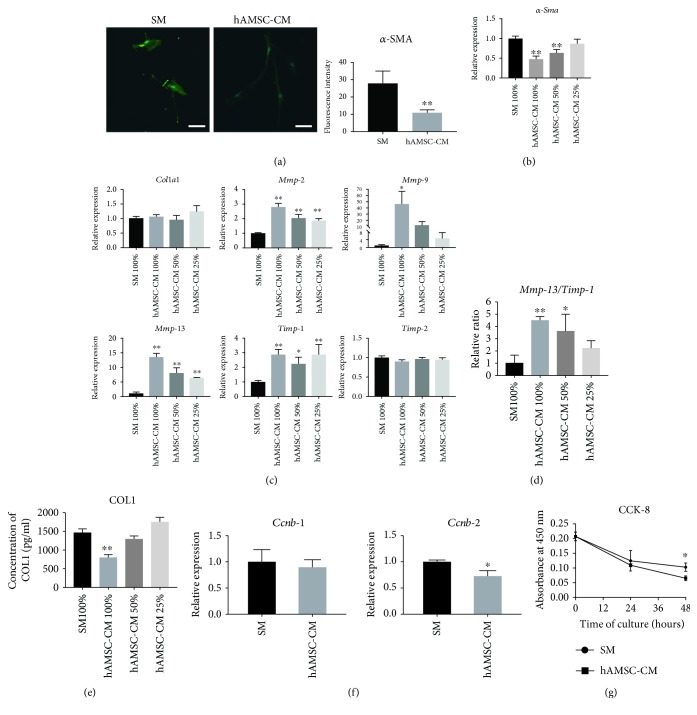
Effect of hAMSC-CM on primary HSC activation and proliferation and ECM accumulation in routine culture. (a) Immunofluorescence staining of *α*-SMA (green) in HSCs cultured in SM or hAMSC-CM for 48 h. Nuclei were stained by Hoechst 33342 (blue). Scale bar = 50 μm. Data are expressed as mean ± SD (*n* = 28 in the SM group and *n* = 24 in the hAMSC-CM group). ^∗∗^ *P* < 0.01 versus SM. (b) Expression of *α-Sma* in different concentrations of hAMSC-CM. Data are expressed as mean ± SD (*n* = 3). ^∗∗^ *P* < 0.01 versus SM 100%. (c) ECM-related gene expression analysis of HSCs cultured in SM or different concentrations of hAMSC-CM. The data are expressed as mean ± SD (*n* = 3). ^∗^ *P* < 0.05 and ^∗∗^ *P* < 0.01 versus SM 100%. (d) Relative *Mmp-13/Timp-1* expression ratio. Data are expressed as mean ± SD (*n* = 3). ^∗^ *P* < 0.05 and ^∗∗^ *P* < 0.01 versus SM 100%. (e) Expression of COL1 in culture media analyzed by ELISA. Data are expressed as mean ± SD (*n* = 3). ^∗∗^ *P* < 0.01 versus SM 100%. (f) Proliferation-related gene expression analysis for *Ccnb-1* and *Ccnb-2*. The data are expressed as mean ± SD (*n* = 3). ^∗^ *P* < 0.05 versus SM. (g) Detection of proliferation of HSCs cultured in SM or hAMSC-CM by CCK-8. The data are expressed as mean ± SD (*n* = 3 for each time point and culture condition). ^∗^ *P* < 0.05 versus SM.

Next, we investigated whether hAMSC-CM inhibits the profibrogenic effects of HSCs, which is a key contributor for fibrosis. After culturing HSCs with hAMSC-CM for 48 h, immunofluorescence staining indicated that *α*-SMA expression in HSCs was much lower compared to HSCs cultured in SM (Figure 3(a)). Consistently, qRT-PCR showed that hAMSC-CM significantly decreased *α-Sma* expression (Figure 3(b)). Then, we examined the expression profile of fibrosis-related genes of HSCs. Compared to control HSCs, hAMSC-CM did not affect *Col1a1* expression (Figure 3(c)). On the other hand, although *Timp-2* expression did not vary, hAMSC-CM significantly upregulated the expression of *Mmp-2*, *Mmp-9*, *Mmp-13*, and *Timp-1* (Figure 3(c)). In addition, hAMSC-CM markedly increased the *Mmp-13/Timp-1* ratio, an index for evaluating the ECM accumulation degree [21] (Figure 3(d)), and decreased the concentration of COL1 in culture media, detected by ELISA (Figure 3(e)). Investigation of the effect of hAMSC-CM on HSC proliferation showed that hAMSC-CM reduced the gene expressions of cyclin B1 (*Ccnb-1*) and cyclin B2 (*Ccnb-2*); however, the reduction of *Ccnb-1* was not statistically significant (Figure 3(f)). The CCK-8 proliferation assay showed that hAMSC-CM significantly inhibited HSC proliferation at 48 h (Figure 3(g)). qRT-PCR and ELISA results indicated that the effect of hAMSC-CM on HSCs was concentration-dependent (Figures 3(b)–3(e)).

### 3.4. Effects of hAMSC-CM on TGF-β-Treated HSCs

**Figure 4:**
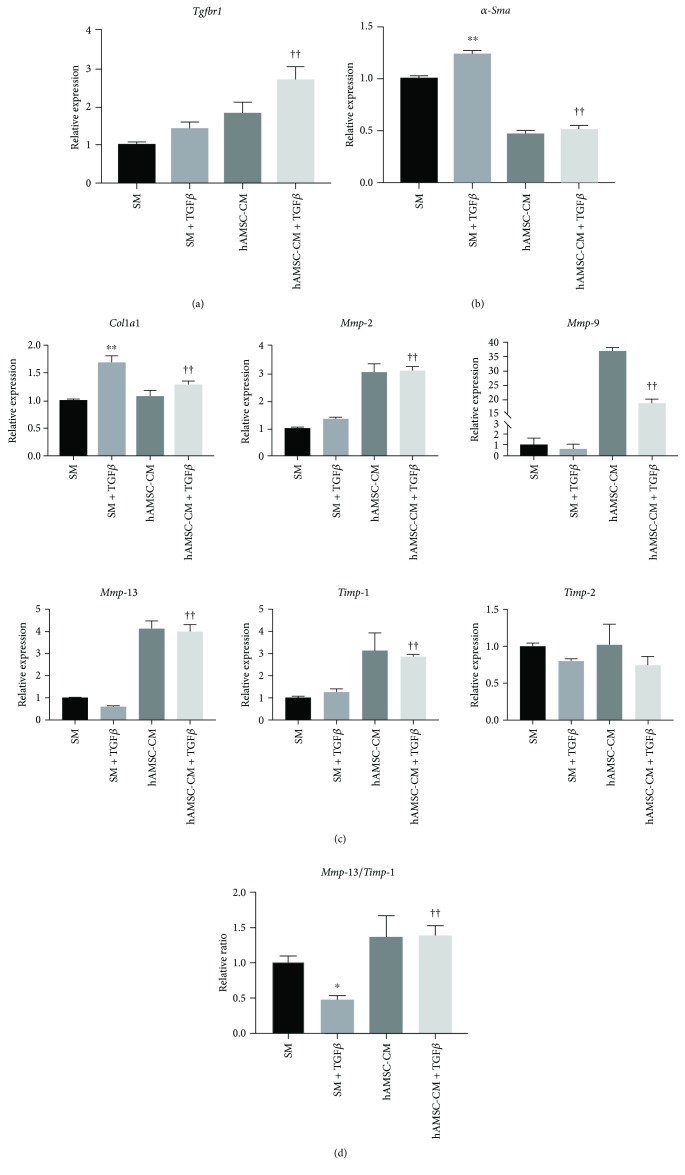
Effect of hAMSC-CM on TGF-β1-induced HSC activation. Gene expression analysis of HSCs with or without TGF-β1 in SM or hAMSC-CM, analyzed by qRT-PCR for (a) *Tgfbr1*, (b) *α-Sma*, and (c) ECM-related genes. (d) Relative *Mmp-13/Timp-1* expression ratio. Data are expressed as mean ± SD (*n* = 3). ^∗^ *P* < 0.05 and ^∗∗^ *P* < 0.01 versus SM; ^††^ *P* < 0.01 versus SM + TGF-β. qRT-PCR, quantitative reverse-transcription polymerase chain reaction.

TGF-β is the most efficient collagen synthesis factor on HSCs [22]; therefore, we investigated whether hAMSC-CM could reverse HSC activation and the progression of ECM accumulation after TGF-β1 stimulation. We observed that TGF-β1 upregulated TGF-β receptor 1 (*Tgfbr1*) expression, and the increased expression of *Tgfbr1* in hAMSC-CM was greater than that in SM (Figure 4(a)). TGF-β1 significantly increased *α-Sma* expression, while hAMSC-CM inhibited the increase in TGF-β1-induced *α-Sma* expression (Figure 4(b)). Although hAMSC-CM did not affect *Col1a1* expression in routine culture, it significantly suppressed TGF-β1-induced upregulation of *Col1a1* (Figure 4(c)). In addition, compared to SM, hAMSC-CM significantly increased *Mmp-2*, *Mmp-9*, *Mmp-13*, and *Timp-1* expression, although there was no change in *Timp-2* expression (Figure 4(c)). TGF-β1 significantly downregulated the *Mmp-13/Timp-1* relative ratio, which was increased, however, by hAMSC-CM, even in the presence of TGF-β1 (Figure 4(d)).

## 4. Discussion

Previous studies have shown that hAMSC transplantation ameliorates liver fibrosis *in vivo* [10, 23]. Given that HSCs play an important role in the development of liver fibrosis [24], we hypothesized that hAMSCs inhibit liver fibrosis by regulating the functions of HSCs with secretory factors. We found that (i) hAMSC-CM inhibits HSC activation, (ii) regulates ECM accumulation during HSC activation, and (iii) suppresses HSC proliferation.

Highly purified HSCs are required for mechanistic studies in liver fibrosis. Therefore, to improve their purity, we isolated HSCs with FACS-based sorting. A previous study has demonstrated that FACS can obtain unaffected, functional HSCs with high purity [15], and we added 7-AAD to prevent interference by nonspecific autofluorescence of dead cells and to ensure that only viable HSCs were sorted. Previous studies reported several markers for HSCs such as desmin [25], glial fibrillary acidic protein (GFAP) [26], and CD38 [27]. However, the specificity of these markers is still questionable [27–29], and desmin staining in the present study showed that only 74.6% were positive (Supplementary Figure 3B). Therefore, we chose using autofluorescence to confirm the purity of isolated HSCs instead of using those markers. In addition, cell type-specific gene expression analysis and flow cytometry analysis indicated that isolated HSCs were rarely mixed with other kinds of cells in the liver (Supplement Figures 3A and 3B). In this study, we obtained highly purified HSCs; however, because of the different amounts of lipid droplets in every cell, FACS may isolate only HSCs full of lipid droplets.

After sorting HSCs, we cultured them in medium with FBS and growth supplement for 48 h to boost HSC adhesion and activation. FBS-free SM or hAMSC-CM was used in the subsequent culture because cytokines and factors present in FBS may mask the potential effects of hAMSC-CM.

HSCs are activated and proliferate rapidly in pathological conditions such as liver injury and transform into myofibroblast-like cells, which express *α*-SMA and secrete abundant collagen [30]. In this study, we demonstrated that hAMSC-CM can inhibit HSC activation, as indicated by the decreased α-SMA expression at both gene and protein levels.

Excessive ECM accumulation, namely, the disequilibrium of interstitial collagens, MMPs, and TIMPs, induces liver fibrosis. When HSCs are activated, large amounts of COL1, the key protein involved in liver fibrosis development, are secreted [31]. In this study, we showed that hAMSC-CM does not influence *Col1a1* expression but has a positive effect on *Mmp*s and *Timp*s in routine culture of HSCs. MMP-13 (the rodent equivalent of MMP-1) is a kind of collagenase and the main protease that can degrade COL1 in a fibrotic liver [30]. Although *Mmp-13* upregulation might imply that hAMSC-CM decreased the amount of COL1, an increase in *Timp-1* made the result indistinct. TIMP-1 is an MMP inhibitor and forms tight 1:1 inhibitory complexes with MMP-13 [32]. Thus, evaluating COL1 degradation with *Mmp-13/Timp-1* is considered more objective [21]. An increase in *Mmp-13/Timp-1* by hAMSC-CM in this study suggested that hAMSC-CM may downregulate the amount of COL1, as verified by a COL1 assay. On the other hand, studies have also reported that MMP-2 (gelatinase A) and MMP-9 (gelatinase B) bind to TIMP-2 and TIMP-1, respectively [33]. Although MMP-2 and MMP-9 barely cleave COL1, their upregulation may also benefit COL1 degradation by blocking of TIMPs. On the basis of the above-mentioned analysis, we believe that instead of inhibiting COL1 synthesis in routine culture of HSCs, hAMC-CM reduces ECM accumulation by promoting COL1 degradation.

TGF-β is the most efficient fibrogenic factor. Stimulated by TGF-β1, upregulation of COL1 and TIMP-1 and downregulation of MMPs lead to ECM deposition [34]. In this study, hAMSC-CM reversed this profibrogenic state and inhibited the increase in TGF-β1-induced *Col1a1* expression, accompanied by *Mmp-2*, *Mmp-9*, and *Mmp-13/Timp-1* ratio upregulation. Although studies have demonstrated that α-SMA expression does not involve the TGF-β signaling pathway [35], the view that TGF-β intensifies α-SMA expression *in vitro* is widely accepted [1, 36]. In this study, we observed that TGF-β1 augments *α-Sma* expression and hAMSC-CM inhibits TGF-β1-induced HSC activation. Interestingly, compared to TGF-β1, hAMSC-CM enhances *Tgfbr1* expression, which appears to be contrary to the antifibrogenic effects of hAMSC-CM. In addition, we found that hAMSC-CM contains TGF-β1 (data not shown), and *Tgfbr1* upregulation is most likely caused by additional exogenous TGF-β1. These results implied that hAMSC-CM exerts antifibrogenic functions by modifying downstream genes in the TGF-β signaling pathway or through the TGF-β-independent pathway. Further studies are required to clarify the underlying mechanism.

HSC activation is accompanied by massive cell proliferation, promoting ECM remodeling and portal resistance increase in liver fibrosis [30]. In this study, the CCK-8 proliferation assay indicated that hAMSC-CM reduces HSC proliferation. In addition, it downregulates the expression of *Ccnb-1* and *Ccnb-2* which are positive cell cycle regulators strongly associated with the G2/M phase. As previous research has clarified that TGF-β1 inhibits cell cycle progression by blocking the activation of cyclin-dependent kinases [37], it appeared that TGF-β1 existing in hAMSC-CM is involved in suppressing HSC proliferation.

Furthermore, in order to investigate whether the suppressive effect on HSCs is specific to hAMSC-CM, we cultured HSCs with CM obtained from skin fibroblasts (fibroblast-CM, Supplementary Figure 4). Although fibroblast-CM significantly enhanced *Col1a1* expression and suppressed *Timp-2* expression in HSCs, it increased the expression of *Mmps* and *Timp-1* and decreased the expression of *α-Sma* and *Ccnb* (Supplementary Figure 4). These results suggest that hAMSCs and fibroblasts have something in common in certain functions. However, because there are few studies demonstrating the similar functions of hAMSCs and fibroblasts, their common mechanism is unclear.

In the present study, we demonstrated the antiactivation effect of hAMSC-CM on HSCs *in vitro*; however, HSCs may display a significant difference *in vivo* [38].

## 5. Conclusion

In conclusion, hAMSC-CM inhibits activation and proliferation of primary HSCs and reduces the accumulation of ECM from HSCs. The results of this study provide mechanistic evidence that hAMSCs play an inhibitory role through paracrine signaling to HSCs. Although several clinical studies report the use of human MSCs in liver fibrosis, the application of MSCs is limited by their availability. Future studies are required to determine the active ingredients and their amounts in hAMSC-CM, which may provide a new approach to treating liver fibrogenesis.

## Figures and Tables

**Table 1:** Sequences of primers.

Gene	Forward primers (5′–3′)	Reverse primers (5′–3′)
*α-Sma*	GACACCAGGGAGTGATGGTT	GTTAGCAAGGTCGGATGCTC
*Col1a1*	GATGGCTGCACGAGTCACAC	ATTGGGATGGAGGGAGTTTA
*Mmp-2*	CTTGCTGGTGGCCACATTC	CTCATTCCCTGCGAAGAACAC
*Mmp-9*	CGCTCATGTACCCCATGTATCA	TCAGGTTTAGAGCCACGACCAT
*Mmp-13*	TCGCATTGTGAGAGTCATGCCAACA	TGTGGTTCCAGCCACGCATAGTCA
*Timp-1*	GACCACCTTATACCAGCGTT	GTCACTCTCCAGTTTGCAAG
*Timp-2*	GGATGGACTGGGTCACAGAG	GCGCAAGAACCATCACTTCT
*Ccnb-1*	CCCTACCAAAACCTGTGGAC	CATCGGAGAAAGCCTGACAC
*Ccnb-2*	TGGAGAGTGAAATACTGGAAGTCA	TGAGAAGCACACGATGGAAG
*Pdgfrb*	GCACCGAAACAAACACACCTT	ATGTAACCACCGTCGCTCTC
*Tgfbr1*	ACCTTCTGATCCATCCGTT	CGCAAAGCTGTCAGCCTAG

## Data Availability

The data used to support the findings of this study are available from the corresponding author upon request.

## Supplementary Materials

Supplementary Materials Supplementary Figure 1: expressions of MSC-specific gene IGTA11 and fibroblast-specific gene CD26 were examined in hAMSCs and skin fibroblasts by quantitative reverse-transcription polymerase chain reaction. Gene expression was normalized to 18s rRNA. Data are shown as mean ± SD (*n* = 3). ^∗∗^ *P* < 0.01 versus hAMSC. Supplementary Figure 2: morphology of sorted HSCs in vitro. (A) Most of the seeded HSCs present an asteroid phenotype with remaining a great quantity of lipid droplets after 1 day of culture. Arrows indicate lipid droplets, and the inset at the upper right corner shows the lipid droplets close-up. Scale bar = 100 μm. (B) Sorted HSCs proliferated during culture and presented a fibroblast-like phenotype. Scale bar = 100 μm. Supplementary Figure 3: characterization of sorted HSCs. (A) Cell type-specific gene expression analysis of sorted HSCs and remaining cells. Beta-type platelet-derived growth factor receptor (Pdgfb) is a marker of HSCs, C-type lectin domain family 4f (Clec4f) is a marker for Kupffer cells, and albumin is a marker for hepatocytes. Data are expressed as mean ± SD (*n* = 3). ^∗^ *P* < 0.05 and ^∗∗^ *P* < 0.01 versus sorted HSCs. (B) Desmin (HSC marker), CD31 (endothelial cell marker), CD163 (Kupffer cell marker) expressions of sorted HSCs were analyzed by flow cytometry. Supplementary Figure 4: gene expressions of primary HSCs cultured in SM or skin fibroblast-CM. Data are expressed as mean ± SD (*n* = 3). ^∗^ *P* < 0.05 and ^∗∗^ *P* < 0.01 versus SM. Supplementary Table 1: sequences of primers.
