# Supplementary material for: Conditioned Medium from Human Amnion-Derived Mesenchymal Stem Cells Regulates Activation of Primary Hepatic Stellate Cells
Source: Stem Cells Int. 2018 Oct 8;2018:4898152. doi: 10.1155/2018/4898152 (PMC6196790; doi:10.1155/2018/4898152)
Supplement: Supplementary Materials — Supplementary Figure 1: expressions of MSC-specific gene IGTA11 and fibroblast-specific gene CD26 were examined in hAMSCs and skin fibroblasts by quantitative reverse-transcription polymerase chain reaction. Gene expression was normalized to 18s rRNA. Data are shown as mean ± SD (n = 3). ∗∗ P < 0.01 versus hAMSC. Supplementary Figure 2: morphology of sorted HSCs in vitro. (A) Most of the seeded HSCs present an asteroid phenotype with remaining a great quantity of lipid droplets after 1 day of culture. Arrows indicate lipid droplets, and the inset at the upper right corner shows the lipid droplets close-up. Scale bar = 100 μm. (B) Sorted HSCs proliferated during culture and presented a fibroblast-like phenotype. Scale bar = 100 μm. Supplementary Figure 3: characterization of sorted HSCs. (A) Cell type-specific gene expression analysis of sorted HSCs and remaining cells. Beta-type platelet-derived growth factor receptor (Pdgfb) is a marker of HSCs, C-type lectin domain family 4f (Clec4f) is a marker for Kupffer cells, and albumin is a marker for hepatocytes. Data are expressed as mean ± SD (n = 3). ∗ P < 0.05 and ∗∗ P < 0.01 versus sorted HSCs. (B) Desmin (HSC marker), CD31 (endothelial cell marker), CD163 (Kupffer cell marker) expressions of sorted HSCs were analyzed by flow cytometry. Supplementary Figure 4: gene expressions of primary HSCs cultured in SM or skin fibroblast-CM. Data are expressed as mean ± SD (n = 3). ∗ P < 0.05 and ∗∗ P < 0.01 versus SM. Supplementary Table 1: sequences of primers. [file 4898152.f1.docx]

***Supplementary Material***

**Conditioned medium from human amnion-derived**

**mesenchymal stem cells regulates activation of primary hepatic stellate cells**

Qingjie Fu, Shunsuke Ohnishi and Naoya Sakamoto

**Correspondence:** Dr. Shunsuke Ohnishi sonishi@pop.med.hokudai.ac.jp

**Figure and Figure Legends**

**Supplementary Figure 1.** Expressions of MSC-specific gene *IGTA11* and fibroblast-specific gene *CD26* were examined in hAMSCs and skin fibroblasts by quantitative reverse-transcription polymerase chain reaction. Gene expression was normalized to *18s rRNA.* Data are shown as mean ± SD (*n* = 3). ^**^*P* < 0.01 versus hAMSC.

**Supplementary Figure 2.** Morphology of sorted HSCs *in vitro*. (A) Most of the seeded HSCs present an asteroid phenotype with remaining a great quantity of lipid droplets after 1 day of culture. Arrows indicate lipid droplets and the inset at the upper right corner shows the lipid droplets close-up. Scale bar = 100 μm. (B) Sorted HSCs proliferated during culture and presented a fibroblast-like phenotype. Scale bar = 100 μm

**Supplementary Figure 3** Characterization of sorted HSCs. (A) Cell type-specific gene expression analysis of sorted HSCs and remaining cells. Beta-type platelet-derived growth factor receptor (*Pdgfb*) is a marker of HSCs, C-type lectin domain family 4f (*Clec4f*) is a marker for Kupffer cells, and *albumin* is a marker for hepatocytes. Data are expressed as mean ± SD (*n* = 3). ^*^*P* < 0.05; ^**^*P* < 0.01 versus sorted HSCs. (B) Desmin (HSC marker), CD31 (endothelial cell marker), CD163 (Kupffer cell marker) expression of sorted HSCs were analyzed by flow cytometry.

**Supplementary Figure 4** Gene expressions of primary HSCs cultured in SM or skin fibroblast-CM. Data are expressed as mean ± SD (*n* = 3). ^*^*P* < 0.05; ^**^*P* < 0.01 versus SM.

**Table**

**Supplementary Table 1** Sequences of primers

| Gene | Forward primers (5’–3’) | Reverse primers (5’–3’) |
| --- | --- | --- |
| *IGTA11*  *CD26*  *18s rRNA*  *Clec4f*  *Albumin* | TCACGGACACCTTCAACATGG  AGTGGCGTGTTCAAGTGTGG  GATATGCTCATGTGGTGTTG  ACGGAGAGCGTGAAGACTGT  TGTCCCCAAAGAGTTTAAAGCTG | CCAGCCACTTATTGCCACTGA  CAAGGTTGTCTTCTGGAGTTGG  AATCTTCTTCAGTCGCTCCA  CTTGCACACCCAGTTGTAGG  TCTTTATCTGCTTCTCCTTGTCTGG |
